# Comparison of the anesthetic efficacy and recovery quality of remimazolam besylate versus sevoflurane for pediatric circumcision: a single-center, prospective, assessor-blinded, randomized controlled study protocol

**DOI:** 10.1186/s12871-025-03378-3

**Published:** 2025-10-28

**Authors:** Yi Zhang, Shuang Guo, Linyun Wang, Qingjun Zeng, Haishan Cui, Yunbo Mo

**Affiliations:** 1 Department of Anesthesiology, Maternal and Child Health Hospital of Wanzhou District, Chongqing City, People’s Republic of China; 2 Nursing Department, Maternal and Child Health Hospital of Wanzhou District, Chongqing City, People’s Republic of China; 3 Department of Pediatrics, Maternal and Child Health Hospital of Wanzhou District, Chongqing City, People’s Republic of China

**Keywords:** Remimazolam besylate, Sevoflurane, Pediatric anesthesia, Circumcision, Anesthetic effect, Recovery quality, Assessor-Blinded trial, Study protocol, Stratified randomization

## Abstract

**Background:**

Administering anesthesia for circumcision in children involves prioritizing safety, comfort, and quality of recovery. While the effectiveness of remimazolam besylate in pediatric anesthesia requires additional confirmation, sevoflurane has been linked to complications like agitation and delirium upon recovery. This protocol describes a planned assessor-blinded randomized controlled trial to evaluate the anesthetic impacts, recovery features, and safety profiles of remimazolam compared to sevoflurane in children between the ages of 3 and 12 undergoing circumcision.

**Methods:**

This single-center, prospective, assessor-blinded, randomized controlled trial will be conducted at the Wanzhou District Maternal and Child Health Hospital. One hundred children (aged 3–12 years, ASA I-II) scheduled for painless circumcision will be randomly allocated to receive either intravenous remimazolam besylate (Group R, *n* = 50) or inhaled sevoflurane (Group S, *n* = 50) for general anesthesia. Both groups will receive a penile root block with 1% lidocaine. Primary outcomes include time to achieve general anesthesia (MOAA/S ≤ 1) and emergence time. Secondary outcomes include anesthesia success rate, intraoperative interventions, hemodynamic changes, respiratory complications, emergence delirium (PAED scale), pain scores (FLACC), and guardian anxiety levels. Due to inherent differences in drug delivery methods, only outcome assessors and data analysts will be blinded. Exploratory subgroup analyses by age (3–6 vs. 7–12 years) will be conducted but are not powered for definitive conclusions. Statistical analysis will use SPSS version 26.0 with significance at *P* < 0.05.

**Ethics and dissemination:**

The Reproductive Ethics Committee at the Wanzhou District Maternal and Child Health Hospital has approved this protocol (Reference No. 2024-44). Consent from the legal guardians will be secured. Findings will be shared via publications in journals and presentations at academic conferences.

**Trial registration:**

This trial has been registered with the Chinese Clinical Trial Registry (ChiCTR) (Registration number: ChiCTR2500095974; Registration date: January 15, 2025).

## Introduction

Circumcision in pediatric patients is a frequently performed surgical intervention to resolve conditions like phimosis, excess foreskin, and associated health concerns, promoting healthy reproductive growth [1]. Given the physiological and psychological underdevelopment in children, it is crucial for anesthesia to prioritize safety and comfort. This involves ensuring smooth induction, maintaining stable vital signs, enabling quick recovery, and providing a clear prognosis while reducing negative effects such as postoperative discomfort, nausea, vomiting, and emergence agitation [2–5]. The effectiveness of anesthesia and the emergence period significantly influence the child’s comfort, the safety of the surgery, the recovery process, and the satisfaction of their guardians.

Remimazolam besylate is a newly created ultra-short-acting benzodiazepine distinguished by a molecular configuration that promotes rapid hydrolysis by non-specific esterases, resulting in an inactive metabolite. This process leads to a brief half-life and clearance that is not reliant on liver or kidney function [6–8]. In comparison to conventional intravenous anesthetics, remimazolam provides a rapid onset, easily adjustable depth of anesthesia, quick and predictable emergence, and exhibits mild effects on respiration and circulation. Furthermore, the sedative effects can be promptly reversed using flumazenil [9, 10]. Its application in sedation for adult diagnostic procedures and minor surgical interventions has been acknowledged, and clinical studies involving children have also indicated its safety and effectiveness for procedural sedation, potentially enabling a better emergence experience [11–14]. However, there remains a necessity for well-designed randomized controlled trials that assess remimazolam’s effectiveness as a primary anesthetic compared to conventional inhalation anesthetics in pediatric patients.

Due to its pleasant scent and quick onset and recovery, sevoflurane is the inhalation anesthetic most frequently utilized in pediatric anesthesia . Although it ensures adequate anesthetic depth and optimal intraoperative conditions for a majority of pediatric procedures, it also presents certain disadvantages. Notably, it can lead to emergence agitation or delirium, a phenomenon particularly common in preschool-aged children [5, 15]. Additionally, it may elevate the likelihood of postoperative nausea and vomiting [4], which undermines the goals of enhanced recovery following surgery.

Pediatric circumcision, recognized as a standard day surgery procedure, necessitates high standards for the speed, efficiency, and quality of anesthetic recovery to facilitate quick recovery and discharge. Given the pharmacological benefits of remimazolam besylate and its positive emergence profile noted in both adult and pediatric research, it is crucial to assess its effectiveness as a primary anesthetic in this context and to compare it with sevoflurane. The fundamental inquiry of this investigation is: does remimazolam besylate, in comparison to sevoflurane, offer superior recovery characteristics while ensuring equivalent anesthetic effectiveness and sustaining similar or improved hemodynamic stability and safety during anesthesia for pediatric circumcision?

Currently, there is a lack of robust randomized controlled trial (RCT) data that directly evaluates and compares the anesthetic effects, recovery quality, and safety of remimazolam besylate and sevoflurane in children between the ages of 3 and 12 undergoing painless circumcision. This study aims to perform a single-center, prospective, randomized controlled trial to thoroughly examine the clinical effectiveness of these two anesthetic methods while ensuring bias is appropriately managed. The design of the study includes stratification by age for subgroup analysis, which will help identify differences in anesthetic outcomes between preschool and school-aged children, thus providing essential data and practical insights for future multicenter confirmatory trials.

## Methods and design

This exploratory clinical trial is single-centered, prospective, randomized, assessor-blinded, and parallel-controlled, adhering to the Declaration of Helsinki [16], CONSORT 2025 [17], and SPIRIT 2025 guidelines [18]. Ethics approval was granted by the Ethics Committee of Wanzhou District Maternal and Child Health Hospital (2024-44).

### Study population

#### Inclusion criteria

1. Boys aged 3–12 years.
2. ASA physical status I or II.
3. Scheduled for circumcision under general anesthesia.
4. No acute upper respiratory tract infection symptoms in the preceding 2 weeks.
5. Written informed consent from a legal guardian.

#### Exclusion criteria

1. Predicted or known difficult airway.
2. Uncontrolled severe systemic conditions.
3. Known allergies to study anesthetics.
4. Personal or family history of malignant hyperthermia.
5. Neuromuscular disorders.
6. Inability to cooperate with clinical evaluations.
7. Guardian refusal to participate.
8. Enrollment in other clinical studies within 30 days (to avoid carry-over effects from other investigational products or interventions, prevent undue research burden on participants and families, ensure that observed outcomes are attributable solely to the interventions of this study, and comply with ethical guidelines for minimizing participant exposure. The 30-day period is a generally accepted washout period to ensure elimination of most investigational drugs, typically > 5 half-lives for many compounds, and to allow for recovery from other research-related procedures).

#### Withdrawal criteria

**Fig. 1:**
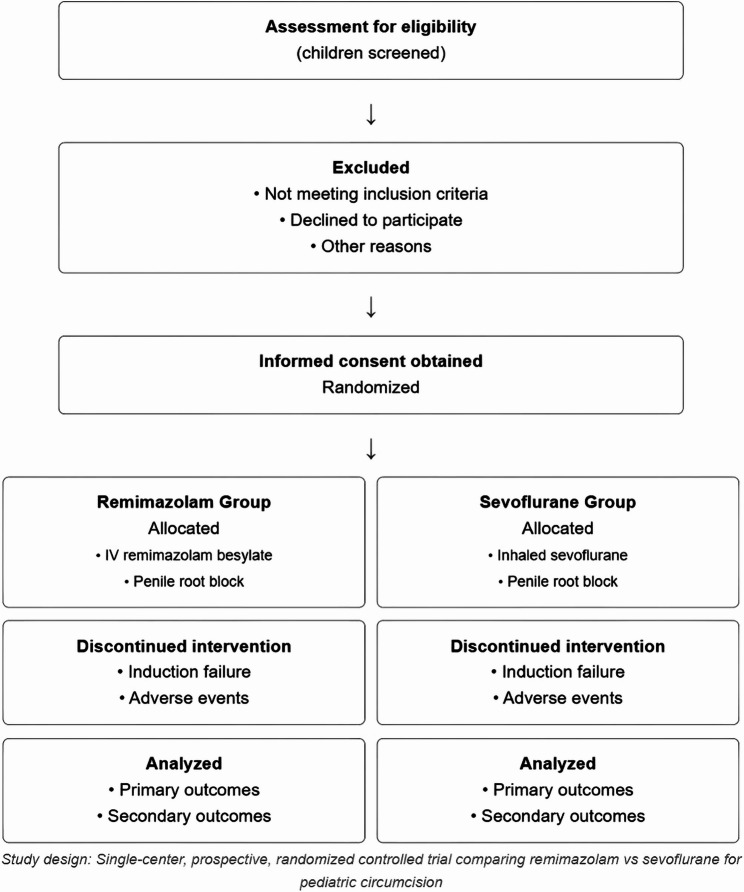
CONSORT flow diagram

Guardian request, serious adverse events, protocol violation, anesthesia induction failure, or significant non-compliance (Fig. 1).

### Randomization and blinding

This is an assessor-blinded trial. We acknowledge that blinding of the anesthesia provider is not feasible due to the fundamental differences in drug delivery methods (intravenous versus inhalation). However, to minimize potential implementation bias, we have made significant efforts and mandated several procedural safeguards. These include: (1) using standardized drug preparation methods; (2) utilizing identical equipment for both groups, including syringes, vaporizers, breathing circuits, and face masks for oxygen delivery; and (3) applying an olfactory confounding technique (consistent application of mint-flavored lip balm for all participants) to mask the scent of sevoflurane. Although we recognize that achieving complete blinding of the anesthesiologist is extremely difficult, we hope to reduce the probability of unblinding and minimize bias through these efforts. We transparently acknowledge this as a significant methodological limitation.

Participants will be randomized 1:1 using computer-generated stratified block randomization (block size of 4), stratified by age (3–6 years and 7–12 years). To ensure blinding, outcome assessors (PACU nurses, research assistants) and data analysts will remain unaware of group allocation.

### Intervention measures

#### Preoperative preparation

Patients will follow standard fasting guidelines. Monitoring will include ECG, NIBP, SpO₂, and ETCO₂. Baseline vital signs, MOAA/S scores, modified Yale Preoperative Anxiety Scale (mYPAS), and guardian anxiety levels (STAI-S) will be documented. Guardian anxiety levels will also be assessed postoperatively to assess for changes and potential correlation with child outcomes. Rationale: Guardian anxiety can significantly affect a child’s cooperation during induction and emergence characteristics through emotional contagion. High parental anxiety often correlates with increased child anxiety, difficult induction, and altered emergence or pain perception.

### Baseline anxiety assessment protocol

All children will undergo baseline anxiety assessment using the modified Yale Preoperative Anxiety Scale (mYPAS) in the preoperative holding area, administered by trained research personnel blinded to group allocation. The mYPAS assessment will be repeated immediately before entering the operating room. Any child with mYPAS score > 30 (indicating significant anxiety) will be documented, and this will be included as a covariate in our sensitivity analyses.

### Anesthetic protocol

Our target is general anesthesia, defined as MOAA/S ≤ 1 [19].

#### Group R (Remimazolam, n = 50)

Induction with remimazolam 0.3 mg/kg IV. Maintenance with remimazolam infusion at 0.75 mg/kg/h (adjustable range 0.2–1.5 mg/kg/h).

#### Group S (Sevoflurane, n = 50)

Induction with 8% sevoflurane via face mask. Maintenance with sevoflurane 1.5–2.5% MAC.

#### Local anesthesia (Both Groups)

A penile root nerve block with 1% lidocaine (max 4 mg/kg) will be administered once MOAA/S ≤ 1 is achieved.

### Anesthesia management and rescue protocol

#### Initial assessment

MOAA/S score is evaluated 120 s after the initial drug dose. If MOAA/S > 1, a rescue dose is permitted within this window.

#### Rescue protocol

If MOAA/S > 1 at 120 s (despite an initial dose), a single rescue dose is given (remimazolam 0.1 mg/kg; max total dose 0.4 mg/kg for remimazolam OR sevoflurane 4–6%). Only ONE rescue dose is permitted during induction for the purpose of this primary outcome assessment. The rescue dose is administered immediately if MOAA/S > 1 at the 120-second assessment point. The maximum total dose for remimazolam (0.4 mg/kg including initial and one rescue dose) and the single rescue dose limit are based on published pediatric and adult safety data and aim to balance efficacy with minimizing the risk of excessive sedation or adverse events.

#### Timeline for induction assessment

1. Time 0: Initial dose administration.
2. Time 120s: MOAA/S assessment → If > 1, give rescue dose.
3. Time 180s: Final assessment (60s after rescue dose).

#### Induction failure

Defined as MOAA/S > 1 at 60 s after the rescue dose. The 120-second window for initial assessment (including a rescue dose if needed) allows for the peak effect of the initial drug administration and one rescue attempt. The subsequent 60-second window after a rescue dose is to prevent prolonged ineffective attempts if the chosen regimen is failing.

#### Intraoperative events

Hypotension (MAP decrease > 20% from baseline) is treated with an initial crystalloid bolus (10 mL/kg over 10–15 min). If persistent, ephedrine 0.05–0.1 mg/kg IV (max 5 mg per dose) will be administered. Rationale: Ephedrine provides indirect sympathomimetic action (inotropic and chronotropic support) and has a favorable pediatric safety profile for anesthesia-induced hypotension. Intraoperative movement is assessed continuously by the anesthesiologist throughout the procedure. Movement requiring intervention is defined as any movement that interferes with surgery or the sterile field, necessitating additional anesthetic administration. MOAA/S is used for induction and emergence assessments, not for continuous intraoperative monitoring of movement. PONV is treated with ondansetron 0.1 mg/kg IV (max 4 mg). If PONV persists or recurs, rescue antiemetics such as dimenhydrinate 0.5 mg/kg IV (max 25 mg) or dexamethasone 0.15 mg/kg IV (max 4 mg) can be considered, depending on prior antiemetic administration and clinical context. No routine prophylaxis is planned due to the short nature of the procedure, but this will be at the discretion of the anesthesiologist if risk factors are high.

### Observation indicators and data collection

#### Primary outcomes

1. Time to general anesthesia (minutes from drug administration to MOAA/S ≤ 1).
2. Emergence time (minutes from drug discontinuation to MOAA/S = 5).

#### Secondary outcomes

1. Rate of anesthesia success (MOAA/S ≤ 1 within 120 s from the start of induction, rescue allowed).
2. Intraoperative movement rate.
3. Hemodynamic changes (MAP/HR at key timepoints).
4. Highest PAED score [20] in PACU. The PAED scale will be assessed by trained PACU nurses who have undergone standardized training with inter-rater reliability testing (kappa > 0.8). Assessment timepoints: arrival to PACU (0 min), then at 5, 10, 15, 20, 25, 30 min, and at discharge. If the child shows signs of agitation (PAED ≥ 12) at 30 min, assessments will continue every 10 min until resolution or discharge. The highest score observed d
  Management of Emergence Delirium: If PAED score ≥ 12 is observed, the following stepwise approach will be implemented:
    1. First-line: Reassurance and parental presence (if institutional policy permits);
    2. Second-line: If agitation persists > 5 min, administer low-dose propofol 0.5-1 mg/kg IV;
    3. Third-line: If severe agitation threatens patient safety, consider rescue midazolam 0.05 mg/kg IV (max 2 mg);
    4. Continuous monitoring until PAED score < 12 for at least 10 min.
5. FLACC score [21] upon awakening (defined as MOAA/S = 5) and then every 10 min while in PACU until discharge. Assessment continues until the score is consistently < 4 or PACU discharge criteria are met. Intervention for pain (e.g., rescue analgesia) will be guided by FLACC score ≥ 4.
  Pain Management Protocol: For FLACC score ≥ 4:
    1. First-line: Assess adequacy of penile block; if > 30 min since block, administer rescue analgesia with acetaminophen 15 mg/kg PO/PR (max 1000 mg);
    2. Second-line: If pain persists after 20 min, administer ibuprofen 10 mg/kg PO (max 400 mg);
    3. Third-line: For severe pain (FLACC ≥ 7), consider low-dose fentanyl 0.5 µg/kg IV (max 25 µg);
    4. Document all interventions and reassess every 10 min until FLACC < 4.
6. Incidence of delirium (PAED score ≥ 12) and other adverse events.
7. PACU stay duration.
8. Guardian anxiety levels (STAI-S) (preoperatively and postoperatively) [22]
9. Guardian satisfaction (VAS scale).

Table 1:Research process and evaluation schedule.

**Table 1:** Research process and evaluation schedule

Study Period/Activity	Screening/Enrollment (-t1)	Pre-anesthesia/Baseline (T0)	Anesthesia Induction (T0→T1)	Intraoperative (T1, T2, ongoing)	Anesthetic Discontinuation (T3)	Emergence/Awakening (T4)	PACU (Post-T4)	Postoperative/Discharge
PROCEDURES/ASSESSMENTS								
Eligibility Screening	O							
Informed Consent (Guardian)	O							
Child Assent (> 7 years)	O							
Randomization (Stratified)	O							
Record Demographics, ASA Status	O							
Guardian Anxiety (STAI-S) - Pre		O						
Fasting Confirmation		O						
IV Line Placement		O						
Standard Monitoring (ECG, NIBP, SpO2, ETCO2)		O	O	O	O	O	O	
Preoxygenation (3 min)		O						
Baseline Vitals & MOAA/S (T0)		O						
Modified Yale Preoperative Anxiety Scale (mYPAS)		O						
INTERVENTIONS								
Group R: Remimazolam Induction			O					
Group R: Remimazolam Maintenance				O				
Group S: Sevoflurane Induction			O					
Group S: Sevoflurane Maintenance				O				
MOAA/S (for sedation onset - T1)			O					
Rescue Sedation/Anesthesia (if needed)			(O if req)	(O if req)				
Penile Root Block (1% Lidocaine)				O (at MOAA/S ≤ 1)				
Anesthetic Discontinuation (T3)					O			
OUTCOME MEASURES								
Time to Sedation Onset (Primary)			Collect					
MAP/HR (T0, T1, T2, T3, T4)		O	O	O	O	O		
Intraoperative Movement/Intervention				Record if any				
MOAA/S (for recovery - T4)						O		
Time to Recovery (Primary)						Collect		
FLACC Score (at awakening (T4) & q10min in PACU until discharge or score < 4)						O	O	
PAED Score (PACU: Multiple timepoints, see protocol)							O	
Incidence of Delirium (PAED ≥ 12)							O	
Adverse Events (Perioperative)		Record if any	Record if any	Record if any	Record if any	Record if any	Record if any	
PONV							Record if any	Record if any
PACU Stay Duration							Collect	
Aldrete Score (for PACU discharge)							O	
Guardian Anxiety (STAI-S) - Post								O
Guardian Satisfaction (VAS)								O
Data Entry into CRF/EDC	O	O	O	O	O	O	O	O

Research process and evaluation schedule. This table Outlines the schedule of all research interventions, data collection points and participant evaluations from screening to postoperative assessment. PAED score assessed in PACU upon arrival (0 min), then at 5, 10, 15, 20, 25, 30 min. If PAED score ≥ 12 at 30 min, assessments continue every 10 min until resolution or discharge. Final assessment at PACU discharge

Abbreviation: *ASA* American Society of Anesthesiologists, *CRF* Case Report Form, *ECG* Electrocardiogram, *EDC* Electronic Data Acquisition System, *ETCO2* End-expiratory carbon dioxide, * FLACC* Scores for facial expression, leg movement, body movement, crying, and soothing situations, *HR* Heart rate, *IV* Intravenous injection, *MAP* Mean Arterial Pressure, *MOAA/S* Modified Observer Alertness/Sedation Score, *NIBP* Non-invasive Blood Pressure, *PACU* Post-Anesthesia Recovery Room, * PAED* Restlessness Score during the Recovery period of Pediatric Anesthesia, *PONV* Postoperative nausea and vomiting, *SpO2* Peripheral blood oxygen saturation, *STAI-S* State-Trait Anxiety Questionnaire (State Section), * VAS* Visual Analogue Scale, *mYPAS* Modified Yale Preoperative Anxiety ScaleTime point definition, *T0* before induction, *T1* MOAA/S ≤1 (sedation begins/anesthesia begins), *T2* When cutting the skin, *T3* When the anesthetic drug stops, * T4* when the patient is conscious (MOAA/S = 5)

### Sample size

A total of 100 participants (50 per group) will be recruited. While our total sample size of 100 participants (50 per group) provides >90% power to detect a 3-minute difference in emergence time (our primary outcome), this results in only ~25 participants per age subgroup (3-6 years vs 7-12 years). This is grossly insufficient for meaningful or definitive subgroup comparisons, which are strictly exploratory. For instance, to detect a clinically significant difference in a binary outcome like emergence delirium rates (e.g., from 35% to 15%), a much larger sample size per subgroup (estimated >70) would be required. Age-stratified subgroup analyses (3-6 years vs 7-12 years) are strictly exploratory with <30% power to detect even large differences in secondary outcomes within subgroups and cannot support any definitive conclusions.

### Data collection and management

Data will be collected using Case Report Forms (CRFs), which may be in either paper format or through an Electronic Data Capture (EDC) system, and will include all relevant variables for the study. Assigned team members will carefully complete the CRFs according to Standard Operating Procedures (SOPs). The Principal Investigator (PI) or a quality control monitor will verify the accuracy and integrity of the data. Electronic data will be encrypted, securely stored, and periodically backed up. Any data inconsistencies will be resolved according to established protocols. Before finalizing the database, a blinded review will be conducted by an independent data manager in collaboration with the PI/statistician. Documentation will be kept for at least five years to adhere to Good Clinical Practice (GCP) standards.

### Data and safety monitoring board (DSMB)

A separate Data Safety Monitoring Board (DSMB), consisting of three unbiased specialists in the fields of anesthesiology, pharmacology, and statistics, will be formed. This DSMB will conduct regular evaluations of safety data (which includes serious adverse events) as well as efficacy information, examine the risk/benefit ratio, and offer guidance to the Principal Investigator (PI) and the sponsor concerning the continuation, alteration, or cessation of the study, all while prioritizing participant safety and adhering to regulatory standards.

### Statistical analysis

Data will be analyzed using SPSS 26.0 with P<0.05 as the significance threshold. The primary efficacy analysis will be on an intention-to-treat basis. Sensitivity analyses will include: (1) adjustment for baseline mYPAS scores as a continuous covariate; (2) subgroup analysis by baseline anxiety level (mYPAS≤30 vs >30); (3) per-protocol analysis excluding any protocol violations. These analyses will help assess the robustness of our findings despite the absence of standardized premedication.

## Discussion (expected)

This protocol describes an assessor-blinded RCT comparing remimazolam and sevoflurane for general anesthesia in pediatric circumcision. This study aims to provide valuable data on the efficacy and safety of remimazolam in this common pediatric procedure.

Should the research validate that remimazolam besylate achieves anesthesia efficacy and maintains intraoperative vital sign control comparable to sevoflurane in pediatric anesthesia, while exhibiting superior onset and emergence times or a decreased frequency of emergence delirium (particularly in preschool-aged children), as well as a similar or reduced rate of perioperative adverse events, its clinical adoption and advocacy will be justified. These findings have the potential to enhance the perioperative experience for children, reduce parental concerns, improve the efficiency of the PACU, and optimize the use of medical resources. Furthermore, if subgroup analysis reveals a notable benefit of remimazolam for preschool children who are vulnerable to behavioral issues, this would guide future studies aimed at precision validation.

Should the findings fail to demonstrate a clear advantage of remimazolam compared to sevoflurane, or if both agents exhibit distinct pros and cons (for instance, excelling in certain metrics while performing less favorably in others), or if safety issues with remimazolam come to light, this research will still retain its scientific importance. This could imply that medical professionals should approach the use of remimazolam with greater scrutiny, or suggest that sevoflurane continues to be a viable and established option. Furthermore, unfavorable results are essential in safeguarding against the misappropriation of medical resources and in reducing potential hazards.

Irrespective of the results, this research will deliver clinical application data regarding remimazolam besylate within pediatric anesthesia, highlighting its pharmacological properties in a targeted demographic. The methodological difficulties (including the execution of blinding) and hands-on experiences (such as dose assessment) encountered in the study will provide valuable information for forthcoming investigations. Limitations of the study (such as its single-center design, small sample size and blinding limitations) will be thoroughly addressed in the report, and the conclusions will be approached with caution. It is anticipated that this research will establish a foundation for future multicenter clinical trials and aid in refining clinical practices.

### Anticipated challenges and limitations

Our most significant limitation is the inability to blind the anesthesiologist to the allocated intervention due to the different administration routes of remimazolam (intravenous) and sevoflurane (inhalation). This could introduce implementation bias, despite efforts to standardize other aspects of care. Another major limitation is the absence of standardized preoperative anxiolytic medication, which may lead to differential baseline anxiety between groups and confound emergence outcomes. Finally, the study is critically underpowered for subgroup analyses, and these results must be interpreted as hypothesis-generating only. Readers must not overinterpret subgroup analyses as these are severely underpowered. The single-center design also limits generalizability.

Despite these limitations, our study design incorporates several strengths including stratified randomization, comprehensive outcome assessment using validated tools, and transparent reporting of all methodological constraints. These features will provide valuable preliminary data to inform future definitive trials.

### Clinical implications and future directions

This study will provide valuable data on the use of remimazolam in pediatric anesthesia. If remimazolam demonstrates a superior recovery profile (e.g., faster emergence, lower incidence of delirium), it could enhance pediatric day surgery practices by potentially improving PACU throughput and patient/family satisfaction. Regardless of the outcome, the findings will be important for informing clinical practice and guiding future research. Future research should incorporate standardized premedication (such as oral midazolam 0.5 mg/kg or intranasal dexmedetomidine 2 µg/kg) administered to all participants to minimize anxiety-related confounding and better reflect best practices for pediatric anxiolysis. Furthermore, larger, multi-center trials adequately powered for subgroup comparisons will be necessary to confirm any exploratory findings from this study and to provide more definitive evidence. This exploratory trial will help establish a foundation for such larger studies.

### Trial status

Protocol version 2.0 (June 25, 2025). Recruitment is planned from July 1, 2025, to June 30, 2026. The study is currently in the protocol finalization phase.

## Data Availability

No datasets were generated or analysed during the current study.
